# Потенциально опасные для отечественного картофелеводства карантинные виды и патотипы нематод: изменчивость популяций и генетика устойчивости картофеля

**DOI:** 10.18699/VJ20.665

**Published:** 2020-11

**Authors:** N.V. Mironenko, T.A. Gavrilenko, A.V. Khiutti, O.S. Afanasenko

**Affiliations:** All-Russian Research Institute of Plant Protection, Pushkin, St. Petersburg, Russia Institute of Cytology and Genetics of Siberian Branch of the Russian Academy of Sciences, Novosibirsk, Russia; Institute of Cytology and Genetics of Siberian Branch of the Russian Academy of Sciences, Novosibirsk, Russia Federal Research Center the N.I. Vavilov All-Russian Institute of Plant Genetic Resources (VIR), St. Petersburg, Russia; All-Russian Research Institute of Plant Protection, Pushkin, St. Petersburg, Russia Institute of Cytology and Genetics of Siberian Branch of the Russian Academy of Sciences, Novosibirsk, Russia; All-Russian Research Institute of Plant Protection, Pushkin, St. Petersburg, Russia Institute of Cytology and Genetics of Siberian Branch of the Russian Academy of Sciences, Novosibirsk, Russia

**Keywords:** potato, parasitic nematodes, Globodera, Ditylenchus, Nacobbus, Meloidogyne, pathotypes, population variability, resistance cultivars, resistance genes, QTL, картофель, паразитические нематоды, Globodera, Ditylenchus, Nacobbus, Meloidogyne, патотипы, изменчивость популяций, устойчивость сортов, гены устойчивости, QTL

## Abstract

Обзор посвящен проблеме потенциально опасных для отечественного картофелеводства каран- тинных видов и патотипов нематод. Картофель поражают более 30 видов паразитических нематод, однако в статье основное внимание уделено самым вредоносным, приносящим большой ущерб картофелеводству пред- ставителям родов Globodera, Ditylenchus, Nacobbus и Meloidogyne. Проанализированы фитопатологические и молекулярные методы идентификации видов и патотипов и основные достижения в изучении изменчивости популяций паразитических нематод картофеля. Показано, что, благодаря особенностям жизненного цикла не- матод и лабильности их геномов, генетическая изменчивость этих организмов очень велика, что создает угрозу образования новых патогенных генотипов паразита. Сведения о внутри- и межпопуляционной изменчивости нематод важны для изучения путей интродукции и распространения отдельных видов, а также поиска корреля- ций молекулярных маркеров с определенным патотипом. Филогенетические исследования, основанные на со- временных данных по генетической изменчивости популяций, позволили выявить комплексы видов у Globodera pallida (Stone) Behrens и Nacobbus aberrans (Thorne) Thorne & Allen (sensu lato), включающие криптические виды. К основным составляющим успешной защиты, предотвращающей массовое распространение паразитических нематод, относятся карантинные мероприятия, агротехнические приемы, биологические способы защиты и возделывание устойчивых сортов. Особое внимание в обзоре уделено вопросам селекции сортов картофеля с длительной устойчивостью к различным видам нематод, поскольку возделывание таких сортов – экологически наиболее безопасный и экономически выгодный способ предотвращения эпифитотий. В настоящее время до- стигнуты значительные успехи в генетической защите сортов картофеля, особенно в отношении цистообразую- щих нематод. Приведены сведения об источниках устойчивости картофеля к паразитическим нематодам, выде- ленных в коллекциях диких и культурных видов. Проанализированы данные об идентифицированных R-генах и QTL устойчивости, которые были интрогрессированы в селекционный материал с помощью различных методов и подходов. Представлены результаты изучения структурной и функциональной организации генов устойчиво- сти к цистообразующим нематодам картофеля. Рассмотрены результаты исследований по использованию моле- кулярных маркеров определенных генов в маркер-опосредованной селекции для создания новых устойчивых сортов, в том числе с групповой устойчивостью.

## Abstract (en)

The review considers quarantine species and nematode pathotypes potentially dangerous for domestic potato production. Potatoes are affected by more than 30 types of parasitic nematodes, but the review focuses on the most harmful representatives of genera that cause great damage to potato production: Globodera, Ditylenchus, Nacobbus and Meloidogyne. Phytopathological and molecular methods of identification of species and pathotypes and the main achievements in studying the population variability of parasitic potato nematodes were analyzed. It was shown that due to the peculiarities of the life cycle of nematodes and lability of their genomes, the genetic variability of these organisms is very high, which creates a threat of forming new pathogenic genotypes of the parasites. The information about the intra- and interpopulation variability of nematodes is important for studying the ways of introduction and distribution of separate species, as well as for searching for the correlations of molecular markers with the pathotype. Phylogenetic studies based on modern data on genetic variability of populations have allowed to reveal species complexes in Globodera pallida (Stone) Behrens and Nacobbus aberrans (Thorne) Thorne & Allen (sensu lato), including cryptic species. The main components of successful protection preventing a wide distribution of parasitic nematodes are quarantine measures, agricultural techniques, biological methods of protection and cultivation of resistant cultivars. Special attention in the review is paid to the breeding of potato cultivars with durable resistance to various nematode pathotypes, because the cultivation of such varieties is the most ecologically safe and economically advantageous way to prevent epiphytoties. Currently, significant progress has been made in the genetic protection of potato cultivars, especially against cyst-forming nematodes. The review provides data on sources of potato resistance to parasitic nematodes identified in collections of wild and cultivated species. Data on identified R-gens and QTL of resistance that have been introduced into breeding varieties using different methods and approaches are analyzed. The literature data on the study of structural and functional organization of genes for resistance to potato cyst nematodes are given. The results of molecular research on revealing the polymorphisms of loci involved in the control of resistance to cyst and gall nematodes, the development of molecular markers of certain genes and their use in marker-assisted selection for developing of new resistant cultivars, including those with group resistance, are considered.

## Введение

Картофель (Solanum tuberosum L.) является одной из ос- новных сельскохозяйственных культур и возделывается на более чем 19 млн га сельскохозяйственных угодий по всему миру c ежегодным производством более 390 млн тонн картофеля (FAOSTAT, 2019). По валовому сбору картофеля Российская Федерация занимает третье место в мире после Китая и Индии, и его среднегодовое про- изводство в стране составляет примерно 29 млн тонн в год. Основной фактор, снижающий рентабельность возделывания картофеля, – болезни, способом контроля которых продолжают оставаться химические средства защиты. Возделывание сортов картофеля с длительной устойчивостью к болезням – экологически наиболее безопасный и экономически выгодный способ предотвращения эпифитотий.

Паразитические нематоды повсеместно приносят большой ущерб производству картофеля, ежегодные глобаль- ные потери урожая от них составляют 10–15 % и оцени- ваются в 78 млрд долларов (Fábia et al., 2018). Картофель поражают 34 вида паразитических нематод, 6 из которых, по крайней мере, могут вызывать значительные потери урожая. Среди них распространенными во всех зонах воз- делывания картофеля и наиболее вредоносными являются цистообразующие картофельные нематоды (ЦКН) – зо- лотистая картофельная нематода (ЗКН) Globodera rostochiensis (Woll.) и бледная картофельная нематода (БКН) Globodera pallida (Stone). Эти виды считаются объектами внешнего и внутреннего карантина и широко распростра- нены на всех континентах: в Европе, Северной и Южной Америке, Азии, Африке, Океании (EPPO, 2020a, b), ЗКН распространена также и в Австралии (Blacket et al., 2019).

Кроме ЦКН, существенные потери урожая картофеля повсеместно могут вызывать карантинные, широкоспециализированные виды нематод, на настоящий день отсутствующие на территории Российской Федерации. В соответствии с единым перечнем карантинных объектов Евразийского экономического союза (ЕАЭС) от 30 ноября 2016 г. № 158 с изменениями и дополнениями от 8 августа 2019 г. в перечень карантинных вредных организмов, от- сутствующих на территории ЕАЭС, входят: ложная гал- ловая нематода Nacobbus aberrans (Thorne) (EPPO, 2019), колумбийская корневая галловая нематода Meloidogyne chitwoodi Golden et al., корневая галловая нематода M. enterolobii Golden et al. и ложная колумбийская галловая нематода M. fallax Karssen (https://docs.eaeunion.org/docs/ru-ru/01413200/cncd_06032017_158). Стеблевая нематода картофеля Ditylenchus destructor (Thorne) относится к ка- рантинным видам в Европейском союзе (список А2), но из-за широкого распространения в нашей стране входит в список А3 – регулируемых некарантинных вредных организмов на территории Российской Федерации (EPPO, 2019b; EPPO, 2020d; OEPP/EPPO, 2017a).

Вопросы классификации видов паразитических кар- тофельных нематод еще не приведены к единой системе. Согласно классификации, принятой EPPO (EPPO, 2000), виды нематод родов Globodera, Ditylenchus, Nacobbus и Meloidogyne, рассмотренных в этом обзоре, относятся к одному классу Chromadorea, и порядку Rhabditida, но к четырем различным семействам: Heteroderidae (виды: Globodera rostochiensis, G. pallida – цистообразующие нематоды), Anguinidae (Ditylenchus destructor – стеблевая нематода картофеля), Pratylenchidae (Nacobbus aberrans – ложная галловая нематода) и Meloidogynidae (Meloidogyne chitwoodi, M. fallax – галловые нематоды). Кроме перечисленных видов рода Meloidogyne, рассматриваются также другие виды этого рода, сведения об устойчивости к которым видов рода Solanum имеются в литературе: M. javanica (Treub, 1885), M. hapla Chitwood, 1949, M. arenaria Chitwood (1949), M. incognita Kofoid & White, 1919.

В литературе обсуждается возможность существования криптических видов у ЦКН (Thevenoux et al., 2019) и видо- вых комплексов, как, например, у G. pallida (Subbotin et al., 2011) и N. aberrans (Cabrera-Hidalgo et al., 2019). На фило- генетическом древе, построенном на основании сходства нуклеотидных последовательностей малой субъединицы рДНК, филум Nematoda разделен на 12 клад, из которых 4 включают нематоды, паразитирующие на растениях. Наиболее вредоносные виды нематод, приводящие к значительным экономическим потерям в картофелевод- стве, – представители четырех родов: Meloidogyne spp., Globodera spp., Ditylenchus spp. и Nacobbus spp., отнесены к кладе Tylenchomorpha (Holterman et al., 2006). Развитие исследований по таксономии паразитических нематод необходимо, прежде всего, для понимания эволюционных процессов в популяциях патогенов, связанных с решени- ем проблемы генетической защиты. Например, в работе R. Thevenoux с коллегами (2019) показано, что генетиче- ские расстояния между географическими популяциями G. pallida на территории Перу могут свидетельствовать о дивергенции популяций с возможным образованием нового вида.

Составляющие успешной защиты, предотвращающей массовое распространение паразитических нематод, – это карантинные мероприятия и возделывание устойчивых сортов. Из существующих биологических способов защи- ты картофеля от паразитических нематод можно отметить использование растений нехозяев, которые стимулируют выход личинок из яиц, но не являются субстратом для их размножения. Таким видом-ловушкой считается, на- пример, Solanum sisymbriifolium (Lam.) (Timmermans et al., 2006). В США (штат Айдахо) использование в каче- стве ловушки S. sisymbriifolium в трех очагах G. pallida позволило снизить жизнеспособность яиц на 95–100 % (Dandurand et al., 2019).

Сорта картофеля с устойчивостью к расширенному спектру патогенов более конкурентоспособны на рынке и обеспечивают получение экологически чистой продукции и сохранение окружающей среды. Последние два фактора имеют не только экономическое, но и существенное со- циальное значение.

Результатом длительной селекции картофеля на устой- чивость как в нашей стране, так и за рубежом стала серия сортов, генетически защищенных от наиболее распро- страненных патотипов цистообразующих картофельных нематод. Например, в списке из 216 сортов картофеля, устойчивых к цистообразующим нематодам, одобрен- ных для использования в ЕС, 213 защищены геном Н1, детерминирующим устойчивость к наиболее распро- страненному, в том числе и на территории РФ, патотипу Ro1 (https://lbst.dk/fileadmin/user_upload/NaturErhverv/Sortsliste_til_internettet_marts_2019.pdf). В России в Гос- реестре селекционных достижений зарегистрировано 455 сортов картофеля, 254 из них устойчивы к ЗКН, т. е. 55.8 % (Государственный реестр селекционных достижений, 2019). При этом необходимо учитывать опыт селекционных программ Великобритании, где во второй по- ловине 20-го века преимущественно возделывались сорта картофеля с геном Н1 устойчивости к ЗКН, что привело к замещению популяций G. rostochiensis на G. pallida (Evans, 1993).

Основная проблема селекции растений на устойчивость к болезням – высокая адаптивная изменчивость патогенов, приводящая к обязательной потере устойчивости при длительном возделывании сортов, защищенных одними и теми же генами, на больших площадях. В отношении ЦКН, и, в частности ЗКН, широкое использование устой- чивых сортов, защищенных геном устойчивости Н1, эффективным против распространенного патотипа Ro1, создает условия для адаптивной эволюции паразита и опасность появления новых патотипов. Не исключен и занос других патотипов ЦКН, а также вида G. pallida с семенным картофелем из Западной Европы. В настоящее время в РФ вид G. rostochiensis представлен исключительно одним патотипом, Ro1, а бледная нематода G. pallida до сих пор не обнаружена. Такая же ситуация существует в Австралии, где распространен только один вид G. rostochiensis и патотип Ro1, а G. pallida не обна- ружен, что было доказано секвенированием ITS участков рибосомных генов (Faggian et al., 2012).

В наше время активно развиваются молекулярно-гене- тические методы видовой диагностики паразитических нематод, появились новые данные по механизмам из- менчивости популяций цистообразующих и галловых нематод, что позволяет определить происхождение мест- ных популяций патогена и прогнозировать их возможные адаптационные изменения к возделываемым сортам картофеля. Имеются успехи в разработке молекулярных маркеров генов устойчивости и создании сортов картофе- ля, генетически защищенных против разных патотипов и видов цистообразующих картофельных нематод. Все эти сведения необходимы для разработки стратегии эффек- тивной генетической защиты против ЦКН в Российской Федерации.

В этом обзоре представлены последние достижения в изучении изменчивости популяций паразитических кар- тофельных нематод, молекулярной диагностики, генетики устойчивости картофеля к нематодам, а также результаты селекции на устойчивость к потенциально опасным для России видам и патотипам цистообразующих и галловых нематод. В статье не затрагивались вопросы биологии фи- топаразитических нематод, хозяино-паразитных отноше- ний, особенностей патогенеза растений, биохимических факторов устойчивости растений и мер контролирования вредоносности нематод, поскольку эти темы подробно анализировались в ряде иностранных обзоров (Sato et al., 2019; Abd-Elgawad, Askary, 2020; Holbein et al., 2020), а также в монографии по фитопаразитическим нематодам (https://www.rfbr.ru/rffi/ru/books/o_1781721#99; Зиновьева и др., 2012).

## Цистообразующие нематоды

Центр происхождения ЦКН – регион Центральных Анд в Южной Америке (Evans et al., 1975; Plantard et al., 2008). Считается, что ЦКН попали в Европу из Южной Аме- рики в середине 19-го века с селекционным материалом картофеля, а оттуда распространились по всему миру. Таким образом, Европа является вторичным центром происхождения ЦКН (Evans, Rowe, 1998; Hockland et al., 2012; Boucher et al., 2013). На территории Российской Федерации широкое распространение получила ЗКН, которая встречается очагами в 7 федеральных округах 61 субъекта РФ на территории общей площадью около 1.8 млн га (Справочник…, 2017). По данным Европейской и Средиземноморской организации по защите растений (ЕРРО), в настоящее время ЗКН распространена во всех странах Европы, в том числе и на территории сопредель- ных стран, таких как Беларусь, Латвия, Эстония, Литва, Украина, Грузия, Армения; на Азиатском континенте в Таджикистане, Японии и Индии (EPPO, 2020a, b). В РФ до настоящего времени БКН не обнаружена (Limantseva et al., 2014; Хютти и др., 2017). В то же время вид G. pallida широко встречается в Европе. По данным ЕРРО, G. pallida зарегистрирована во всех странах Европы, кроме Беларуси, Латвии, Литвы, Польши и Словакии (https://gd.eppo.int/taxon/HETDPA/distribution/RU_ru). В Украине первое обнаружение G. pallida было отмечено в Ужгороде в 2005 г. (Pylypenko et al., 2005), но после карантинных мероприятий уже в 2014 г. в существующем очаге пато- ген не был выявлен, ЕРРО официально зафиксировала отсутствие этого вида для страны. В сопредельных с РФ Европейских государствах G. pallida была выявлена в 2018 и в 2019 гг. в Эстонии (NPPO of Estonia (2018-02, 2018-11, 2019-06)), а также в Норвегии и Финляндии (Holgado, Magnusson, 2012). На Азиатском континенте очаги БКН зафиксированы в Индии и Японии. В связи с боль- шими объемами импорта как продовольственного, так и семенного картофеля в Россию в последние десятилетия существует потенциальная возможность заноса БКН на территорию страны.

Известны еще два вида ЦКН из рода Globodera, пара- зитирующие на картофеле. G. leptonepia (Cobb & Taylor) была обнаружена только однажды в партии картофеля из Южной Америки, но интенсивные поиски в районе Анд- ского Нагорья, известного как центр происхождения ЦКН, не были успешными, таким образом, G. leptonepia – ред- кий и неизученный вид (Thevenoux et al., 2019). В 2008 г. в США в штатах Айдахо и Орегон обнаружены единичные цисты, которые по морфологической и молекулярной диа- гностике не соответствовали ни G. rostochiensis, ни G. pallida (Dandurand et al., 2019). Новый вид был определен как Globodera ellingtonae n.sp. (Handoo et al., 2012) и было показано, что картофель и томаты – его растения-хозяева (Skantar et al., 2011). Отсутствие сведений о распростра- нении этого вида в других регионах мира, по-видимому, свидетельствует о наличии локальной эндемичной по- пуляции на Северо-Американском континенте.

Цистообразующие картофельные нематоды – облигатные специализированные седентарные паразиты, для которых характерно наличие в жизненном цикле цист, со- держащих яйца и инвазионные личинки. Потери урожая могут достигать более 80–90 % (OEPP/EPPO, 2017b). Основной диагностический признак G. pallida – окраска цист: в конце созревания они не приобретают золотистый цвет, как у G. rostochiensis, а остаются бледными. Цисты обоих видов могут выживать в почве без растений-хозяев до 10 лет (Sijmons, 1993; Williamson, Hussey, 1996), однако имеются данные о сохранении их жизнеспособности до 30 лет (Winslow, Willis, 1972). Максимальные потери уро- жая, ассоциированные с поражением ЦКН, определены в 80 % (Brodie, Mai, 1989), но при высокой инфекционной нагрузке ЦКН в почве урожай картофеля может быть полностью уничтожен (Whitehead, Turner, 1998). Четкие симптомы глободероза часто не заметны в период веге- тации картофеля (Lilley et al., 2007). Вследствие этого по- тери урожая могут быть приписаны вторичным болезням, возникшим на уже зараженных нематодой ослабленных растениях картофеля.

Видовая диагностика с применением морфометриче- ских и молекулярных методов широко применяется в практике карантина растений для идентификации видов ЦКН в обнаруженном очаге в посадках картофеля. Мор- фометрические методы диагностики достаточно подробно описаны в коллективной монографии (Зиновьева и др., 2012), а также в обзорах и методических указаниях (Шестеперов, 2002; Ryss, 2002; Bairwa et al., 2017; Christoforou et al., 2017; OEPP/EPPO, 2017b).

## Молекулярные методы идентификации видов ЦКН

Молекулярные методы идентификации видов нематод меняются параллельно с развитием базовых методов молекулярной генетики и зависят от конкретных задач исследователя. В последние годы молекулярную диагности- ку различных видов нематод проводят методами обычной ПЦР, ПЦР в реальном времени (количественная ПЦР) и обратной ПЦР (ОТ-ПЦР). Во всех случаях обычно ис- пользуют праймеры, разработанные к последовательно- стям участков кластера генов рибосомных РНК. Методы идентификации G. rostochiensis и G. pallida, основанные на рестриктном анализе продуктов амплификации фраг- ментов ITS1 или ITS1+5.8S+ITS2 (Thiéry, Mugniery, 1996; Fleming et al., 1998), сменились более удобными методами ПЦР с видоспецифичными праймерами (Bulman, Marshall, 1997; Zouhar et al., 2000; Vejl et al., 2002).

В наших работах для молекулярной идентификации видов G. rostochiensis и G. pallida (Мироненко и др., 2013, 2015; Limantseva et al., 2014) применен метод муль- типлексной ПЦР с двумя наборами видоспецифичных праймеров (Bulman, Marshall, 1997). Эти же праймеры используют другие авторы, например, для количественной ПЦР (Nakhla et al., 2010).

Для оценки эффективности методов деконтаминации растительного материала от ЦКН определяют жизнеспо- собность цист, применяя ОТ-ПЦР как индикатор экспрес- сии генов «домашнего хозяйства», например гена gpd1, кодирующего глицерол-3-фосфат-дегидрогеназу – одного из основных ферментов гликолиза (Kaemmerer, 2012). В этой работе виды нематод были определены предвари- тельно по методу S.R. Bulman и J.W. Marshall (1997) с по- мощью стандартной ПЦР, так как ОТ-ПЦР не может быть использована для идентификации видов нематод, пока не будут обнаружены видоспецифичные гены, необходимые для выживания организма, аналогичные генам «домашне- го хозяйства». Вероятно, полногеномное секвенирование обоих видов нематод (Cotton et al., 2014; Eves-van den Akker et al., 2016) сделает доступной информацию для выявления таких генов.

## Патотипы G. rostochiensis и G. pallida

Оба вида ЦКН могут быть классифицированы на патоти- пы на основании их реакций вирулентности/авирулент- ности на дифференцирующих тест-клонах картофеля с различными генами устойчивости (табл. 1) (Canto Saenz, De Scurrah, 1977; Kort et al., 1977). Для G. rostochiensis были определены пять патотипов, Ro1–Ro5; для G. pallida – три, Pa1–Pa3 (Kort et al.,1977).

**Table 1.**
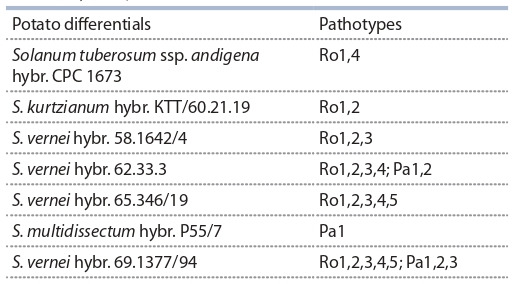
Set of potato differentials for identifying pathotypes of cyst nematodes (Canto Saenz, De Scurrah, 1977; Kort et al., 1977)

В 1990 г. дифференциация популяций ЦКН была пере- смотрена на основании нечетких различий между пато- типами, определяемыми на данном наборе, что связано с различной жизнеспособностью инокулюма и наличием количественной частичной устойчивости, уровень кото- рой трудно интерпретировать (Nijboer, Parlevliet, 1990). По мнению авторов, основанном на анализе литературы и собственных экспериментальных данных, возможно идентифицировать только три патотипа: Ro1 (бывшие Ro1 и Ro4), Ro3 (бывшие Ro2 и Ro3) и Ro5 (бывший Ro5). Клоны-дифференциаторы 60.21.19 и 65.346.19 различают только виды нематод, но не их патотипы. В отношении G. pallida H. Nijboer и J.E. Parlevliet (1990) считают воз- можным характеризовать популяцию только как более или менее вирулентную, не выделяя патотипы. В то же время в Нидерландах в 1975 г. было отмечено обильное размножение БКН на сортах, устойчивых к патотипу Ра2. По реакции клона-дифференциатора (VTN)’ 62-33-3 изо- лят был отнесен к патотипу Pa3 (Dellaert, Vinke, 1987). Авторами показан различный уровень размножения па- тотипа Ра3 на разных генотипах картофеля.

По мнению Европейской и Средиземноморской органи- зации по защите растений, термин «патотип» в отношении ЦКН считается слишком общим, и многие популяции, основываясь на Международной схеме по дифференциации (Kort et al., 1977), не могут быть адекватно иден- тифицированы и отнесены к тому или иному патотипу (OEPP/EPPO, 2017b). В настоящее время предлагается проводить изучение вирулентности местных популяций G. rostochiensis и G. pallida на наборах сортов, выращиваемых в той или иной стране, со следующими эталонными популяциями ЦКН: Ro1 Ecosse (INRA, Франция), Ro5 Harmerz (BBA, Германия), Pa1 Scottish (SASA, Велико- британия) и Pa3 Chavornay (INRA, Франция). В связи с тем, что в селекции картофеля используются различные генетические источники устойчивости, вирулентность эталонных популяций ЦКН должна регулярно перепро- веряться по отношению к этим наборам сортов. Точно так же, если в результате мониторинга будет обнаружено, что характеристики вирулентности популяций ЦКН нематод в Европе изменились, то эталонные популяции ЦКН следует пересмотреть (OEPP/EPPO, 2006, 2017b).

Во всем мире широко распространен патотип ЗКН Ro1, и, соответственно, большинство устойчивых к ЗКН сортов защищены эффективным против этого патотипа геном Н1. Преодоление этого гена другими патотипами ЗКН зареги- стрировано в Польше, где впервые в 2013 г. был выявлен патотип Ro5 (Przetakiewicz, 2013). В США в течение более 50 лет с момента первого обнаружения ЗКН ген Н1 оста- вался эффективным против распространенного патотипа Ro1. В 1995–1996 гг. на Лонг Айленде и в северной части штата Нью-Йорк на сортах, защищенных геном Н1, было отмечено появление размножающейся популяции ЗКН, которая была определена как патотип Ro2 (Brodie, 1995; 1996). Интересно, что на обширных территориях, напри- мер в Российской Федерации и Австралии (Виктория), встречается только один вид, G. rostochiensis, и один патотип этого вида, Ro1 (Limantseva et al., 2014; Blacket et al., 2019).

Появление патотипа Ра2/3 G. pallida в США в штате Айдахо произошло в результате одной интродукции в 2006 г. Последующие интенсивные исследования показали распространение этого вида на полях общей площадью 369 га, в основном путем механического переноса ино- кулюма с сельскохозяйственной техникой. В связи с этим немедленно было введено эмбарго на импорт картофеля из этого штата традиционным партнерам в Канаду, Южную Корею и Мексику. Япония прекратила импорт картофеля из США (Dandurand et al., 2019). Таким образом, серьез- ный экономический ущерб от появления G. pallida на небольших площадях одного региона был связан главным образом с необходимыми карантинными ограничениями.

## Генетическая изменчивость популяций ЗКН и БКН

Цистообразующие картофельные нематоды группируются в патотипы согласно их способности размножаться на дифференциаторах (Canto Saenz, De Scurrah, 1977; Kort et al., 1977). Идентификация патотипа фитопатологическим методом занимает много времени, поэтому предпринима- ются попытки найти молекулярные маркеры, способные идентифицировать патотипы. Однако до сих пор эти по- пытки были безуспешны.

В работах, посвященных изучению генетической из- менчивости ЦКН, употребляется термин «популяция», которую рассматривают как вариант патотипа. Интересно, что разные популяции одного патотипа можно различить с использованием комбинации молекулярных маркеров, например гена-эффектора rbp-1, некодирующих участков ядерной (ITS регион) и митохондриальной (scmtDNA IV) ДНК (Hoolahan et al., 2012).

В этом плане представляют интерес результаты изуче- ния нуклеотидного полиморфизма гена-эффектора pel-2, который у нематод рода Globodera кодирует эффекторную молекулу пектат-лиазу, участвующую в деградации клеточной стенки растения-хозяина. Однако наблюдаемый полиморфизм не был связан с определенным патотипом G. rostochiensis или G. pallida, хотя были выявлены видо- специфичные сайты (Geric Stare et al., 2011).

В качестве генетического маркера патотипов G. pallida рассматривают также ген-эффектор Gp-Rbp-1 (Carpentier et al., 2012), который кодирует секретируемый белок, индуцирующий специфический, или эффектор-активиро- ванный иммунитет (effector-triggered immunity), опосре- дованный геном устойчивости Gpa2 Solanum tuberosum. Создана база данных из 158 полиморфных последователь- ностей гена Gp-Rbp-1, найдено 8 сайтов, которые находят- ся под контролем положительного естественного отбора. Из них только один сайт, P/S 187, подходит для объяснения узнавания Gp-Rbp-1 геном Gpa2 (Carpentier et al., 2012).

Данные, полученные при анализе внутри- и межпо- пуляционной изменчивости ЦКН, важны для оценки путей интродукции и распространения, а также поиска корреляций с патотипом. Для этого используют различные молекулярные маркеры – RAPD (Blok et al., 2006; Миро- ненко и др., 2015), AFLP (Folkertsma et al., 2001), SSR (Plantard et al., 2008; Boucher et al., 2013), SNP (Davis et al., 2005). Например, с помощью микросателлитных маркеров была оценена изменчивость популяций патотипа Ro1 G. rostochiensis в Австралии (штат Виктория). Показано большое сходство между австралийскими популяциями по частотам аллелей микросателлитных локусов и в то же время отличие их от других популяций, собранных по всему миру. Этот факт свидетельствует об одноразовом заносе ЗКН в Австралию, что дает возможность легко контролировать появление новых видов и патотипов не- матод (Blacket et al., 2019).

В 2011 г. был разработан новый высокопроизводи- тельный метод генотипирования GBS (genotyping-bysequencing) (Elshire et al., 2011). Позднее был внедрен метод Pool-Seq, основанный на секвенировании ДНК сложных образцов, пулов, вместо индивидуумов (Futschik, Schlotterer, 2010). Среди ЦКН распространена полиан- дрия, т. е. множественное оплодотворение самки разными самцами (Turner, Rowe, 2006), поэтому одна циста может содержать несколько сотен генетически различных яиц. Именно пулы цист (по 15 цист) нематод были объектом исследования с помощью комбинации подходов Pool- Seq и GBS, что позволило изучить отношения между 23 популяциями G. rostochiensis из девяти стран, пред- ставляющими пять патотипов (Mimee et al., 2015). На филогенетическом древе, построенном для 23 популяций на основании частот аллелей для 604 SNP-локусов, популяции сгруппировались по генетическому сходству и патотипному составу в два кластера: Ro1, Ro2 и Ro3, Ro4, Ro5. Отмечено большое генетическое сходство двух популяций ЗКН из США, относящихся к разным патоти- пам (Ro1 и Ro2). Авторы считают, что из-за длительного использования устойчивых сортов картофеля с геном H1 развитие вирулентной популяции (Ro2) может быть ре- зультатом недавней мутации, а не интродукции нового патотипа ЗКН (Mimee et al., 2015).

Возможно, в ближайшее время будет разработан новый подход к молекулярной диагностике патотипов Ro1 и Ro2, основанный на использовании результатов полногеномного секвенирования множественных линий Ro1 и Ro2, полученных из одной цисты, и выявлении одно- нуклеотидных полиморфизмов (Dandurand et al., 2019).

Данные литературы по генотипированию популяций нематод с помощью молекулярных маркеров показывают, что один патотип может объединять популяции нематод, различающиеся не только по географическому происхождению, но и генетическим параметрам изменчивости. Например, с использованием комбинации методов Pool-Seq и GBS были получены сведения о генетическом разно- образии популяций, на основании которых рассчитаны индексы фиксации (Fst) при попарном сравнении попу- ляций G. rostochiensis. Так, патотип Ro1 был представлен в работе 14 популяциями из девяти стран: минимальное значение, Fst = 0.04, получено при сравнении популяций из Германии и Бельгии, а максимальное, Fst = 0.43, – для популяций из США и Канады. Можно предполагать, что на фоне такой высокой генетической изменчивости вы- сока вероятность адаптивной эволюции, приводящей к образованию новых патотипов (Mimee et al., 2015).

Адаптация паразита к генотипам хозяина, защищенным эффективными генами устойчивости, может быть связа- на со сниженной фитностью на восприимчивых сортах. «Цена» новой вирулентности состоит в том, что клоны с такой вирулентностью становятся менее агрессивны на сортах с отсутствием комплементарного гена устойчи- вости, т. е. на восприимчивых сортах. Вследствие этого снижается конкурентоспособность таких особей в при- родных популяциях паразитов. Такие факты приведены в обзоре (Laine, Barres, 2013) для грибов, оомицетов, вирусов и нематод, в частности Meloidogyne incognita (Castagnone-Sereno et al., 2007). На примере G. pallida была показана адаптация паразита после 8–10 пассажей на сорте картофеля Iledher с QTL устойчивости GpaV_vrn_, интрогрессированным от Solanum vernei и картированным на хромосоме V (Fournet et al., 2013). Авторы пришли к неожиданному выводу об увеличении фитности линий с новой вирулентностью на восприимчивом сорте Desiree, которая выражалась в формировании увеличенных по размеру цист, содержащих большее количество личинок, и в сокращении времени образования личинок из яиц. Поскольку эти данные были получены в лабораторных условиях, то, безусловно, требуется подтверждение тако- го сценария адаптации G. pallida в условиях природных популяций.

Для понимания эволюционной истории G. pallida про- ведены обширные сборы популяций ЦКН на картофель- ных полях Перу (Picard et al., 2004). Сорок две перуанские популяции G. pallida были проанализированы с использо- ванием восьми микросателлитных маркеров. Популяции разделились на южные и северные и образовали пять кластеров. Большее генетическое разнообразие выявлено на юге Перу. В связи с этим была выдвинута идея о том, что процессы горообразования в Андах вызвали множество адаптивных изменений у нематод и стимулировали про- цесс видообразования у представителей рода Globodera (Grenier et al., 2010). Эти исследования были продолже- ны на более широком материале перуанских популяций G. pallida. Были генотипированы 117 популяций с исполь- зованием данных секвенирования гена cathepsin L, участвующего в процессе питания нематоды и включающего 12 интронов, и нового набора из 13 микросателлитных ло- кусов (Thevenoux et al., 2019). В результате было выявлено гораздо большее генетическое разнообразие популяций, которые были структурированы в шесть групп: 1a, 1b, 2, 3, 4 и “pallida Chilean type”. Оказалось, что генетические расстояния между популяциями G. pallida объясняются не только географическим фактором, но и климатиче- скими условиями, а также типом почвы. Показано, что длина продукта амплификации гена cathepsin L служит диагностическим видоспецифичным маркером для ви- дов G. rostochiensis, G. pallida и G. ellingtonae. Изоляты групп 1, 2 и 3 имеют один и тот же аллель гена cathepsin L, что свидетельствует об их генетической близости и позволяет считать их популяциями одного вида. Популяции группы 4 и группы pallida Chilean type могут считаться криптическими видами внутри видового комплекса G. pallida. О генетической отдаленности группы 4 от остальных говорит факт обнаружения у изолятов этой группы уни- кальной аллели гена cathepsin L. О дивергенции группы pallida Chilean type свидетельствуют высокие значения Fst при сравнении с G. pallida, которые были выше 0.5, что сравнимо для Fst между перуанскими G. rostochiensis и G. pallida (=0.58) и G. pallida и G. mexicana (=0.48) (Thevenoux et al., 2019).

## Генетика устойчивости картофеля к цистообразующим нематодам и перспективы селекции

**G. rostochiensis (ЗКН).** Источниками устойчивости кар- тофеля к различным патотипам ЗКН и БКН служат мно- гие южноамериканские и мексиканские виды картофеля (Castelli et al., 2003; Nunziata et al., 2010; Dalamu et al., 2012). У ряда устойчивых образцов культурных и близко- родственных диких видов картофеля было идентифициро- вано около 25 генетических факторов, детерминирующих устойчивость к ЦКН: R-гены, обеспечивающие реакцию сверхчувствительности (H1, Gpa2) или конститутивно экспрессирующиеся во всех тканях растения (Gro1-4), и локусы количественных признаков (QTL), вовлеченные в контроль длительной олиго- и полигенной устойчивости либо в контроль частичной устойчивости к одному или нескольким патотипам одного из видов нематод (табл. 2). Идентифицированы также два QTL, вовлеченные в контроль частичной групповой устойчивости к разным видам ЦКН, оба картированы на хромосоме V в одном и том же кластере: Grp1_QTL, определяющий устойчивость кар- тофеля к патотипу Ro5 ЗКН и к патотипам Ра2/3 БКН (Finkers- Tomczak et al., 2009, 2011), и Ro2_A QTL устой- чивости к патотипу Ro2 ЗКН и к патотипам Ра2/3 БКН (Park et al., 2019).

Гены, контролирующие устойчивость к наиболее рас- пространенным патотипам ЦКН, были клонированы и секвенированы: ген Gpa2 устойчивости к патотипам Pa2 и Pa3 БКН (Rouppe van der Voort et al., 1997; Van der Vossen et al., 2000), Gro1-4, определяющий специфическую устойчивость растений к патотипу Ro1 ЗКН, входящий в сложный кластер генов семейства Gro1 (Paal et al., 2004), и ген H1 устойчивости к патотипам Ro1/Ro4 ЗКН (Finkers- Tomczak et al., 2011). Выявлена сложная структура этих локусов, содержащих как полноразмерные, так и дефектные копии гомологов R-генов, – RGH (resistance gene homologues). Продукты этих генов относятся к раз- ным классам рецепторных белков, взаимодействующих с эффекторами патогена. Так, ген Gpa2 кодирует белок семейства LZ-NBS-LRR (Van der Vossen et al., 2000), Н1 – белок семейства CC-NB-LRR (Finkers-Tomczak et al., 2011), а Gro1-4 – структурно отличный белок, относящийся к семейству TIR-NB-LRR с дополнительным TIR-доменом, гомологичным Toll-подобному рецептору, активирующему IL-1 (Paal et al., 2004).

Для детекции у растений функциональных аллелей R-генов устойчивости к ЦКН, а также локусов количественных признаков, контролирующих частичную устой- чивость к глободерозам, разработаны многочисленные ДНК-маркеры, которые широко применяются в маркер- опосредованной селекции (MAS) (Dalamu et al., 2012; Хютти и др., 2017), а также мультиплексные системы (Asano et al., 2012), позволяющие существенно повысить эффективность селекционно-генетических программ по созданию устойчивых к ЦКН сортов картофеля.

Как упоминалось выше, наиболее часто нематодоустой- чивые сорта защищены доминантным аллелем гена Н1, со- храняющим уже более 50 лет свою эффективность против наиболее распространенного патотипа ЗКН Ro1 (Ellenby, 1954; Gebhardt et al., 1993). Сорта с генами Gro1-4, или GroV1, или с QTL-локусами серии Gro1 также проявляют устойчивость к патотипу Ro1 G. rostochiensis (см. табл. 2). В то же время в сортиментах сортов и селекционных кло- нов разных стран частота встречаемости образцов с этими генами различна. Так, по данным польских коллег, 77 % из 61 протестированных зарубежных сортов обладали маркерами гена Н1 и 28 % – маркером гена Gro1-4, при- чем результаты молекулярного скрининга коррелировали с данными фитопатологических тестов (Milczarek et al., 2011). Из 812 сортов и селекционных клонов японского селекцентра NARO (Хоккайдо) у 33 % образцов были выявлены маркеры гена Н1, в то же время ни один из протестированных образцов не обладал маркером гена Gro1-4 (Asano et al., 2012). Аналогично в выборке из 58 селекционных клонов индийской селекции около 60 % генотипов имели маркеры гена H1, а генотипов с марке- ром гена Gro1-4 не обнаружено (Sudha et al., 2016, 2019). При этом около 20 % из 217 протестированных образцов коллекции UACh Университета Южного Чили оказались MAS-позитивными в скрининге с маркером гена Gro1-4 (López et al., 2015). Согласно суммарным результатам мо- лекулярного скрининга 225 российских сортов картофеля и сортов ближнего зарубежья (114 из которых входят в Государственный реестр селекционных достижений, до- пущенных к использованию в РФ), от 2 до 5 маркеров гена Н1 были выявлены у 28 % изученных сортов, а маркеры гена Gro1-4 – лишь у 2 % (Антонова и др., 2016; Клименко и др., 2017; Гавриленко и др., 2018). Очевидно, что в се- лекционных программах разных стран использованы разные доноры R-генов устойчивости к патотипу Ro1 G. ro-stochiensis. В цитированных выше работах описано раз- личное число маркеров, но в большинстве случаев в на- боры входили и наиболее информативные из них: Gro1-4-1 гена Gro1-4, а также маркеры TG689 и 57R гена Н1.

**Table 2.**
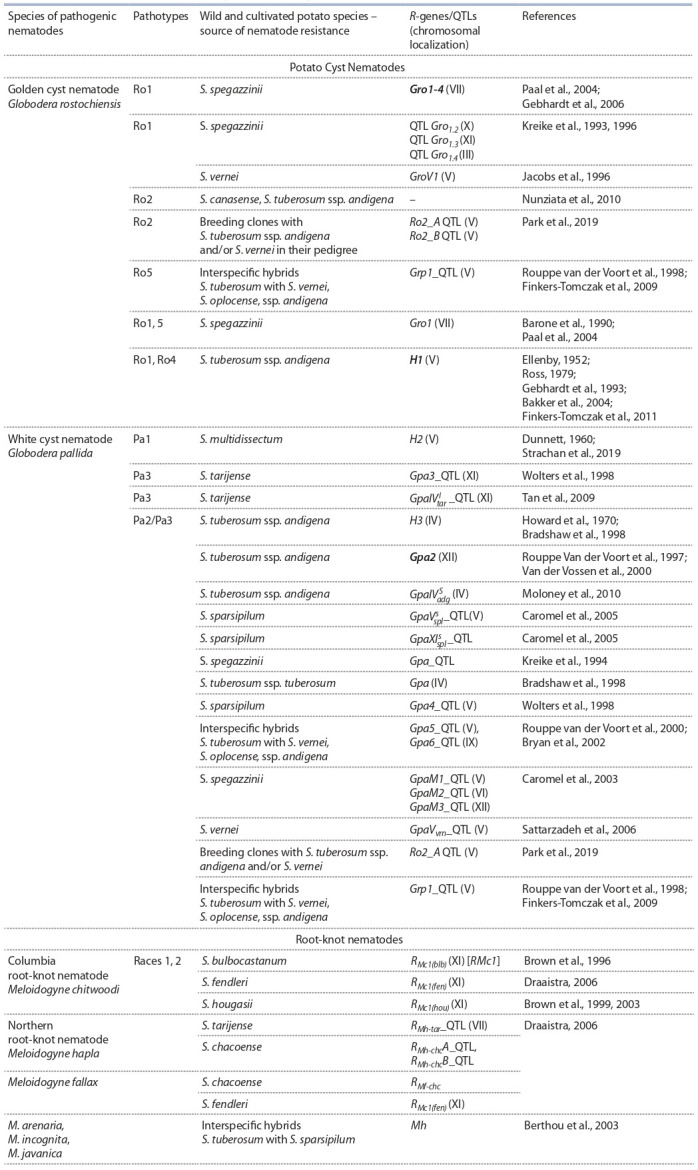
Resistance (R-) genes/QTLs identified in wild and cultivated Solanum species which confer resistance to different pathotypes of potato cyst nematodes and root-knot nematodes Notе: Bold type indicates R-genes that were mapped, cloned and sequenced.

**G. pallida (БКН).** Источники устойчивости картофеля к патотипам Ра2/P3 БКН достаточно широко использованы в селекции. Так, маркеры гена Gpa2 выявлены у 17.5 % образцов выборки из 812 сортов и селекционных клонов японского селекцентра NARO, (Asano et al., 2012); марке- ры гена Gpa2 и/или Gpa5_QTL детектированы у устойчи- вых к БКН зарубежных сортов и отсутствовали у воспри- имчивых (Milczarek et al., 2011). Из 66 клонов индийской селекции примерно у половины были детектированы маркеры локусов GPaVvrn_QTL и Gpa5_QTL устойчивости к Ра2/3 БКН, причем почти все MAS- позитивные геноти- пы были фенотипически устойчивы к БКН (Sudha et al., 2019). Целенаправленный поиск источников устойчивости к G. pallida в нашей стране до последнего времени фак- тически не проводился, поскольку этот патоген в России не обнаружен. С использованием аллель-специфичного маркера гена Gpa2 среди 193 отечественных сортов вы- явлено 24 (12.4 %) MAS-позитивных – потенциальных источников устойчивости к БКН (Гавриленко и др., 2018; Клименко и др., 2019). Особый интерес для последующих селекционных работ, направленных на пирамидирование генов устойчивости к разным вредным организмам, пред- ставляют сорта, сочетающие маркеры генов Gpa2 и H1 устойчивости к обоим видам цистообразующих нематод – G. pallida и G. rostochiensis

Сорта и селекционные клоны картофеля, устойчивые к G. rostochiensis, несущие доминантный аллель гена H1 устойчивости к патотипам Ro1/4, поражаются другими патотипами ЗКН (Brodie, 1995, 1996; Przetakiewicz, 2013), известно также, что растения с маркерами локуса Gpa5 поражаются ЗКН (Sudha et al., 2019). В связи с этим в по- следнее десятилетие усилия исследователей разных стран направлены на поиск источников групповой устойчивости к глободерозам. С этой целью проведены фитопатологи- ческие тесты, по результатам которых среди образцов диких (S. kurtzianum, S. sparsipilum, S. vernei) и культурных видов (S. stenotomum) были отобраны источники устой- чивости ко всем известным патотипам G. rostochiensis (Ro1–Ro5) и G. pallida (Pa1–Pa3) (Dalamu et al., 2012). Такие источники групповой устойчивости были также выделены в различных комбинациях межсортовых скре- щиваний (Przetakiewicz et al., 2017). В селекционных программах США удалось отобрать пять селекционных клонов, одновременно устойчивых к трем видам цистоо- бразующих картофельных нематод: G. rostochiensis (Ro1 и Ro4), G. pallida и G. ellingtonae (Whitworth et al., 2018). Интересно отметить, что большая часть генотипов, устой- чивых и к G. rostochiensis (Ro1 и Ro4), и к G. ellingtonae, обладали маркерами локуса Н1, что позволило авторам предположить наличие у этих клонов нового локуса, тес- но сцепленного с Н1, детерминирующего устойчивость картофеля к G. ellingtonae (Whitworth et al., 2018).

В европейской базе данных по сортам картофеля (The European Cultivated Potato Database, ECPD) приведены списки большого количества сортов с устойчивостью к различным патотипам цистообразующих картофельных нематод.

## Галловые нематоды

Известно более 90 видов галловых нематод из рода Meloidogyne – широко специализированных патогенов, пора- жающих более 2000 видов растений, в том числе карто- фель (Hunt et al., 2009; Moens et al., 2009). Наибольший ущерб картофелеводству галловые нематоды наносят в районах тропического и субтропического климата (Lima et al., 2018). Благоприятными для развития большинства видов рода Meloidogyne являются условия повышенной температуры, наличие ирригации и легкая песчаная почва (Jones et al., 2017). В таких условиях потери от поражения галловыми нематодами, например такими, как M. javanica и M incognita, могут составлять 100 %. В регионах с уме- ренным климатом, таких как Европа и Северная Америка, распространенный и вредоносный на картофеле вид – ко- лумбийская галловая корневая нематода – M. chitwoodi. Этот вид может поражать картофель при температуре ниже 6 °С (Jones et al., 2017).

Галловые нематоды – эндопаразиты. Инфекционными являются личинки второй стадии развития, которые, вы- лупившись из яиц, передвигаются в почве и находят корни растения, а далее с использованием стилета и набора энзимов, проникают в клетки корня и начинают питаться. В результате гипертрофии и гиперплазии соседних клеток образуются гигантские клетки, в которых происходят еще три линьки. Половозрелая самка откладывает в среднем 400–500 яиц между клетками кортикальной паренхимы или на поверхности корней. Первая линька личинок происходит внутри яйца, после чего появляется инфекционная личинка второго возраста. Взрослые самцы не паразити- руют на растении, они выходят в почву и погибают (Lima et al., 2018). Пораженные растения картофеля – низко- рослые, хлоротичные, с бурыми пятнами на листьях и гнилью клубней.

Для галловых нематод характерны различные типы размножения: от классического амфимиксиса до митотического партеногенеза. Для партеногенетических видов Meloidogyne, к которым относятся M. chitwoodi, M. javanica и M incognita, не подходит концепция биологического вида. Идентификация видов построена на определении морфологических признаков и поддержана биохимическими данными, основанными на изоферментном анализе (Dalmasso, Berge, 1978). Для применения этих методов требуются зрелые самки. Последние 30 лет разрабатывали методы молекулярной диагностики галловых нематод на основе ДНК полиморфизмов рибосомной и митохондри- альной ДНК и анонимных локусов, таких как RFLP, RAPD и AFLP (Hyman, Powers, 1991; Powers, 2004). Источни- ками ДНК в этом случае могут быть яйца, личинки или взрослые особи. В настоящее время методы диагностики галловых нематод, как и других, основаны на ПЦР с видо- специфичными праймерами, например для M. incognita и M. javanica (Donkers-Venne et al., 2000; Dong et al., 2001), M. chitwoodi и M. fallax (Wishart et al., 2002).

## Генетика устойчивости картофеля к галловым нематодам и перспективы селекции

На сегодняшний день устойчивые к галловым нематодам селекционные сорта картофеля еще не выведены, однако источники устойчивости к разным видам рода Meloidogyne были выделены среди диких видов, на их основе создаются перспективные селекционные клоны и исследуется генетика устойчивости картофеля к этим видам паразитических нематод. Так, в результате фитопатологического скрининга на устойчивость к галловым нематодам более 5000 индивидуальных растений 64 диких ви- дов картофеля показано, что устойчивые к M. chitwoodi и M. fallax образцы встречаются в основном среди центрально- и североамериканских видов (Janssen et al., 1996), у которых был установлен моногенный контроль устой- чивости (Brown et al., 1989, 1991). Многие диплоидные виды этих регионов принадлежат к третичному генпулу и из-за барьеров презиготической несовместимости не могут скрещиваться с S. tuberosum. Интрогрессия гена R_Mc1(blb)_, обеспечивающего резистентность диплоидного мексиканского вида S. bulbocastanum к расам 1 и 2 ко- лумбийской галловой корневой нематоды M. chitwoodi, была осуществлена с помощью межвидовой соматической гибридизации с S. tuberosum. В результате косегрегационного анализа популяций, полученных в возвратных скре- щиваниях этих соматических гибридов, проведенного с использованием хромосомспецифичных RFLP-маркеров, ген R_Mc1(blb)_ был картирован на хромосоме XI (Brown et al., 1996).

Ортологичные гены устойчивости к M. chitwoodi – R_Mc1( fen)_ и R_Mc1(hou)_ – идентифицированы у аллополипло- идных мексиканских видов Solanum hougasii и S. fendleri (Brown et al., 1999, 2003, 2009; Draaistra, 2006) (см. табл. 2). В серии работ C.R. Brown с коллегами (1996, 1999, 2003, 2009, 2014; Zhang et al., 2007) получены интрогрессивные формы картофеля поколений ВС_5_–ВС_6_ с генами R_Mc1(blb)_, R_Mc1( fen)_ и R_Mc1(hou)_ диких мексиканских видов, а также разработаны сцепленные с ними STS- и CAPS-маркеры для проведения маркер-опосредованной селекции. Устой- чивость интрогрессивных форм обусловлена генерацией активных форм кислорода и активацией защитных меха- низмов, в которых центральную роль играет салициловая кислота. Она запускает реакции сверхчувствительности и каскада физиологических и биохимических реакций, предотвращающих развитие M. chitwoodi в корнях рас- тений (Bali et al., 2019). Важно отметить, что клоны с идентифицированным геном R_Mc1( fen)_ устойчивости к M. chitwoodi одновременно обладали и устойчивостью к M. fallax (Draaistra, 2006).

Среди южноамериканских диких видов картофеля образцов, устойчивых к M. chitwoodi и к M. fallax, не об- наружено, за исключением единичных образцов у трех видов: S. berthaultii, S. chacoense, S. gourlayi. По резуль- татам AFLP-анализа гибридной популяции S. tuberosum × S. chacoense, был идентифицирован QTL RMf-chc устой- чивости к M. fallax (Draaistra, 2006).

Многочисленные источники устойчивости к северной галловой нематоде M. hapla были отобраны среди как северо-, так и южноамериканских видов (Janssen et al., 1996). Постзиготическая несовместимость, проявляющая- ся в большинстве комбинаций межвидовых скрещиваний диких видов Южной Америки с S. tuberosum, относитель- но легко преодолевалась использованием дигаплоидов сортов картофеля или экспериментальных полиплоидов диких диплоидных видов для достижения значений «эффективной плоидности» (EBN), а также подбором совместимых комбинаций скрещиваний (Гавриленко, Ермишин, 2017).

Таким образом, устойчивые к M. hapla образцы диких видов Южной Америки относительно легко вовлека- лись в гибридизацию с S. tuberosum; расщепляющиеся гибридные популяции использовали для картирования локусов, обуславливающих частичную устойчивость к M. hapla (Draaistra, 2006). В этой работе на хромосоме VII был картирован R_Mh-tar__QTL дикого аргентинского вида S. tarijense, два других QTL, R_Mh-chc_ A и R_Mh-chc_ B, так и не удалось картировать из-за нарушений колинеарно- сти хромосом S. chacoense и S. tuberosum, однако были отобраны AFLP-маркеры, ассоциированные с локусами устойчивости к северной галловой нематоде (Draaistra, 2006) (см. табл. 2).

Важным практическим результатом работ по картированию R-генов и QTL устойчивости картофеля к галловым нематодам считается разработка сцепленных с ними ДНК-маркеров (Rouppe van der Voort et al., 1999; Draaistra, 2006), которые применяются в маркер-опосредо-ванной селекции. Следует отметить, что первые резуль- таты исследований по пирамидированию R_Mh-chcA__QTL и R_Mh-tar__QTL оказались нерезультативными, поскольку сре- ди клонов, отобранных в MAS, не выявлено устойчивых к M. hapla (Tan et al., 2009). Исследования, направленные на поиск эффективных комбинаций QTL-локусов и R-генов для создания высокоустойчивых к галловым нематодам генотипов, продолжаются.

В работе F. Berthou с коллегами (2003) изучен характер наследования устойчивости к различным видам галловых нематод гибридов ди- и тетраплоидных культурных ви- дов картофеля с близкородственным южноамериканским видом S. sparsipilum. Результаты анализа девяти гибрид- ных комбинаций (из F_1_, F_2_ и возвратных скрещиваний) позволили установить моногенную устойчивость к трем видам галловых нематод, M. arenaria, M. incognita, M. javanica, обусловленную доминантным аллелем гена Mh (см. табл. 2). Вместе с тем отобранные устойчивые гено- типы поражались M. mayaguensis, M. chitwoodi, M. fallax, M. hapla. Важно отметить, что устойчивость гибридов, определяемая геном Mh, существенно снижается при повышении температуры от 24 до 29 °C (Berthou et al., 2003), что необходимо учитывать при проведении фитопатологических тестов в регионах с тропическим и субтропическим климатом, где широко распространены галловые нематоды.

## Виды паразитических нематод картофеля, генетика устойчивости к которым не изучена

**Стеблевая нематода картофеля** Ditylenchus destructor (Nematoda, Tylenchida) – широко распространенные виды в регионах с умеренным климатом в Европе, в том числе в России, Северной Америке, Азии, Океании и Южной Африке (Mai et al., 1990; Шестеперов и др., 2006, 2010; Sigareva et al., 2012; OEPP/EPPO, 2017а).

Вредоносность стеблевой нематоды картофеля D. destructor состоит в повреждениях клубней, которые по- крываются растрескивающимися темными пятнами, при этом симптомы поражения надземной части растения отсутствуют. Весь жизненный цикл нематод длится около шести дней и проходит в клубнях картофеля, в которые они проникают через кожуру около глазков, питаясь крах- мальными зернами (Mai et al., 1990)

Стеблевая нематода D. dipsaci поражает стебли, столоны и клубни картофеля. На клубнях образуются серовато- бурые пятна, растение отстает в росте. Пораженные стебли изгибаются и становятся вздутыми. Источник инфекции – пораженные клубни (Lima et al., 2018). Многочисленные исследования генетической изменчивости популяций D. dipsaci показали, что этот вид фактически является ком- плексом видов (Subbotin et al., 2005). Для идентификации видов Ditylenchus были разработаны видоспецифичные универсальные праймеры к последовательностям генов и спейсерным участкам генов рибосомального кластера, дававшие в ПЦР специфический продукты амплифика- ции: D. destructor – 1200 п. о., D. dipsaci и D. myceliophagus – 900 п. о. (Vrain et al., 1992). Для идентификации нормальной и гигантской рас D. dipsaci использовали ПЦР с применением SCAR-праймеров (Esquibet et al., 2003); праймеры на 5.8S рДНК были разработаны для мо- лекулярной диагностики D. destructor (Marek et al., 2005). Протоколы по молекулярной диагностике подробно опи- саны EPPO – European and Mediterranean Plant Protection Organization (OEPP/EPPO, 2017а). Видоспецифичные праймеры на ITS-участки были использованы для иденти- фикации D. destructor в Московской области (Mahmoudi et al., 2019). В литературе были сообщения об устойчивых и толерантных к D. destructor и D. dipsaci сортах картофеля (Mwaura et al., 2015). Генетика устойчивости картофеля к клубневой и стеблевой нематодам не изучена.

**Ложная галловая нематода** Nacobbus aberrans – эндемичный вид для Южно-Американского континента, тем не менее из-за широкого диапазона хозяев, включающего 84 вида растений, и экономического значения для таких культур, как картофель, томат и сахарная свекла, она от- несена к карантинным объектам. Для картофеля потери урожая составили в среднем 65 % в Андском регионе Ла-тинской Америки. Таксономические проблемы рода Nacobbus рассмотрены в обзоре (Manzanilla-López, 2010). Современные молекулярные данные позволили присвоить N. aberrans статус видового комплекса. Для обнаружения Nacobbus spp. в почве и клубнях картофеля используют методы RFLP и секвенирование ITS-rDNA-областей (Reid et al., 2003), ПЦР с видоспецифичными праймерами (Atkins et al., 2005). В литературе сообщалось об источниках устойчивости к Nacobbus aberrans, отобранных среди об- разцов диких южноамериканских видов: S. microdontum, S. acaule, S. okadae (Franco, Main, 2006), генетика устойчи- вости картофеля к ложной галловой нематоде не изучена.

## Заключение

В настоящее время защита картофеля от паразитических нематод сводится к двум основным составляющим: 1) ка- рантинным мероприятиям, которые не всегда эффективны из-за иногда неконтролируемого перевоза посадочного материала и сельскохозяйственной техники между част- ными хозяйствами, и 2) использованию устойчивых к нематодозам сортов, в том числе с групповой устойчиво- стью к разным видам нематод. Вследствие этого поиск для селекции новых генов устойчивости, в том числе эффек- тивных против потенциально опасных патотипов и видов паразитических нематод, создадут «подушку безопасно- сти» в случае их заноса на территорию РФ или появления в результате адаптационных процессов. Информация о картированных R-генах и QTL устойчивости к нематодам и ассоциированных с ними ДНК-маркерах существенно повышает эффективность селекционно-генетических про- грамм по созданию новых сортов, устойчивы

Перспективным представляется развитие новых моле- кулярных технологий. Последние 20 лет с момента обна- ружения РНК интерференции (RNAi) (Fire et al., 1998), эту технологию пытаются использовать для защиты растений от различных видов нематод (Lilley et al., 2007; Bairwa et al., 2017). Показано, что экспрессия в растении-хозяине двуцепочечной РНК, нацеленной на гены домашнего хо- зяйства или паразитизма у галловой нематоды, приводит к устойчивости против этой нематоды (Gheysen, Vanholme, 2007). Дальнейшее развитие молекулярных технологий, а также исследований биологии паразитических нематод картофеля, изменчивости популяций, эволюции, струк- турной и функциональной геномики как паразитов, так и их хозяев позволит совершенствовать способы контроля заболеваний картофеля, вызываемых паразитическими нематодами.

## Conflict of interest

The authors declare no conflict of interest.
